# Fraction B From Catfish Epidermal Secretions Kills Pancreatic Cancer Cells, Inhibits CD44 Expression and Stemness, and Alters Cancer Cell Metabolism

**DOI:** 10.3389/fphar.2021.659590

**Published:** 2021-07-19

**Authors:** Jassim M. Al-Hassan, Daoyan Wei, Sharmistha Chakraborty, Tara Conway, Patrea Rhea, Bo Wei, Megan Tran, Mihai Gagea, Mohammad Afzal, Sosamma Oommen, Divya Nair, Bincy M. Paul, Peiying Yang

**Affiliations:** ^1^ Department of Biological Sciences, Faculty of Science, Kuwait University, Kuwait City, Kuwait; ^2^ Department of Gastroenterology, Hepatology and Nutrition, The University of Texas MD Anderson Cancer Center, Houston, TX, United States; ^3^ Department of Palliative, Rehabilitation and Integrative Medicine, The University of Texas MD Anderson Cancer Center, Houston, TX, United States; ^4^ Department of Veterinary Medicine and Surgery, The University of Texas MD Anderson Cancer Center, Houston, TX, United States

**Keywords:** catfish, Fraction B, pancreatic cancer, cancer stemness, apoptosis, cancer metabolism

## Abstract

Pancreatic ductal adenocarcinoma (PDAC) is the fourth leading cause of cancer related death in western countries. The successful treatment of PDAC remains limited. We investigated the effect of Fraction B, which is a fraction purified from catfish (*Arius bilineatus, Val.*) skin secretions containing proteins and lipids, on PDAC biology both *in-vivo* and *in-vitro*. We report here that Fraction B potently suppressed the proliferation of both human and mouse pancreatic cancer cells *in vitro* and significantly reduced the growth of their relevant xenograft (Panc02) and orthotopic tumors (human Panc-1 cells) (*p* < 0.05). The Reverse Phase Protein Array (RPPA) data obtained from the tumor tissues derived from orthotopic tumor bearing mice treated with Fraction B showed that Fraction B altered the cancer stem cells related pathways and regulated glucose and glutamine metabolism. The down-regulation of the cancer stem cell marker CD44 expression was further confirmed in Panc-1 cells. CBC and blood chemistry analyses showed no systemic toxicity in Fraction B treated Panc-1 tumor bearing mice compared to that of control group. Our data support that Fraction B is a potential candidate for PDAC treatment.

## Introduction

Pancreatic cancer (PC) is one of the most aggressive cancers of the digestive system (Siegel et al., 2020). According to the WHO, adenocarcinoma is the most common type of PC and pancreatic ductal adenocarcinoma (PDAC) is currently the third leading cause of cancer-related death in United States. It is expected to surpass colorectal cancer and become the second by the year 2030 (Siegel et al., 2020)**.** Pancreatic cancer patients have a dismal prognosis with less than 8% survival rate for five years and less than 6 months average survival duration from diagnosis (Siegel et al., 2020). The diagnosis and treatment of PDAC is currently an important medical problem and cancer associated mortality is primarily caused by early metastasis and therapeutic resistance or recurrence. Cancer stem cells (CSCs), a subpopulation of chemo-resistant/radioresistant, self-renewable, and multipotent cells in the bulk of the tumor, are associated with the high malignancy of PDAC. These cells can initiate tumor and cause rapid tumor growth, resistance to therapy, recurrence, and metastasis (Sneddon and Werb, 2007; Merlos-Suárez et al., 2011; Oskarsson et al., 2014). CSCs have been found in several types of neoplasms. Specific targeting of CSCs therapeutically provides hope for improving survival and quality of life for PDAC patients.

CD44 is expressed as a wide variety of isoforms in many cells. It is the major hyaluronan (HA) receptor and is involved in cell adhesion, migration, homing, proliferation, and differentiation (Naor et al., 2008; Yan et al., 2015). CD44 is encoded by 20 exons and undergoes extensive alternative splicing to generate CD44 standard (CD44s) and CD44 variant (CD44v) forms. CD44s consists of the first five exons (exons 1–5) and the last five exons (exons 16–20), while CD44v is generated by including either one or multiple variable exons between the exon 5 and exon 16. The CD44 variable exons are numbered v1 to v10 (v1 is not encoded in human), which correspond to the genomic exons 6–15, respectively (Yan et al., 2015)**.** Accumulating evidence shows that CD44, especially its CD44v isoforms, are CSC markers that play critical roles in regulating the properties of CSCs, including self-renewal, tumor initiation, metastasis, and chemo/radio resistance (Yan et al., 2015; Skandalis et al., 2019). Accordingly, therapies that target CD44 may destroy the CSC population leading to the cure of life-threatening cancers (Oskarsson et al., 2014; Li et al., 2018; Xiao et al., 2018). In human pancreatic cancer, the levels of CD44s were significantly higher in cancer tissues than adjacent nontumor tissues, and patients whose tumors expressed high levels of CD44s had shorter survival duration than those with low levels (Li et al., 2014). Administration of Anti-CD44s antibody reduced growth, metastasis, and post radiation recurrence of pancreatic xenograft tumors in mice (Li et al., 2014). The antibody also reduced the number of tumor-initiating cells (TICs) in cultured pancreatic cancer cells and xenograft tumors, as well as their tumorigenicity (Li et al., 2014). Alternatively, injection of the v6 peptides that block the co-receptor functions of CD44v6 for MET and VEGFR-2 inhibited human pancreatic xenograft tumor growth and metastasis and decreased survival of KPC mice (Matzke-Ogi et al., 2016). Interestingly, almost all recurrent human PDAC tumor cells became CD44^+^ following a standard chemotherapy for PDAC (Molejon et al., 2015a). The recurrent human PDAC-derived xenograft tumors that were irresponsive to gemcitabine treatment became sensitive to an anti-CD44 monoclonal antibody-based therapy in mice (Molejon et al., 2015b). These results suggest that CD44 represents an important therapeutic target in patients with PDAC. Accordingly, a number of CD44 targeted strategies have been entered into clinical trials (NCT03009214, NCT01358903, NCT02254005, NCT02254031). The available clinical trial results are not satisfactory as we expected (Molejon et al., 2015b), while more clinical trials are still ongoing. Therefore, continuing search for novel therapeutic approaches that can specifically target CSCs for PDAC treatment remain extremely important.

FDA approval of anticancer drugs that originated from natural products amounted to 41% of the total approved drugs. This indicates the importance of natural products as sources of new therapeutic agents (Newman and Cragg, 2012; Cragg and Pezzuto, 2016). The catfish (*Arius bilineatus, Val.*) elaborates gel-like material through its epidermis when stressed. The secretion is composed of biologically active lipids and proteins (Al-Hassan et al., 1982; Al-Hassan et al., 1986a; Al-Hassan et al., 1987). Preparations from the epidermal secretions of the catfish (CSP) have previously been shown to affect the vascular system (Wang M. M. et al., 2001), blood clotting and accelerate healing of wounds in animal and in man and healing of non-healing diabetic foot ulcers in man (Al-Hassan et al., 1983; Al-Hassan et al., 1985; Al-Lahham et al., 1987; Al-Hassan, 1990; Al-Hassan et al., 1991; Summers et al., 1991; Al-Hassan et al., 1998). The preparations have also been found to exert various biological activities, such as smooth muscle contraction (Al-Hassan et al., 1986b) and increase in vascular permeability in rat skin (Wang M. M. et al., 2001). Al-Hassan and his colleagues demonstrated that furanoic acid or F-6, a C-20 fatty acid (12,15-epoxy-13,14-dimethyleicosa-12,14-dienoic acid), is present in the lipid fraction (Ft-3) of CSP (Al-Hassan et al., 2019). They established that the lipid mixture (Ft-3) isolated from CSP and its F-6 component dose dependently inhibited human leukemic K-562 and human breast cancer MDA MB-231 cells (Al-Hassan et al., 2019). Fraction B is derived from CSP (Al-Hassan, 2013; Al-Hassan et al., 2020; Ali, 2020). It contains both lipids and proteins. However, whether Fraction B which contains Ft-3 can inhibit the growth of pancreatic cancer has yet to be determined.

The objective of this study was to define whether Fraction B has tumor inhibitory action on pancreatic cancer and if yes, what are the molecular mechanisms involved. The results of our study demonstrated that Fraction B has potent antitumor activity in pancreatic cancer, mainly through inhibiting pancreatic CSC marker CD44 expression and altering critical tumor cell metabolic pathways with no, or very limited systemic toxicity, suggesting the high translational value of Fraction B for pancreatic cancer therapy.

## Materials and Methods

### Materials

All procedures were performed according to the relevant guidelines, rules and regulations of The University of Texas MD Anderson Cancer Center. Fraction B of catfish skin secretion preparation (CSP) was prepared by Dr. Jassim M. Al-Hassan and his group at Kuwait University utilizing the procedures described in the previously published two patent applications (Patent No. 10,568,915 B1 and Patent No. 8,551,532 B2) (Al-Hassan, 2013; Ali, 2020). Anti-CD44, Anti-CD133, Anti-Met and Anti-GAPDH were purchased from Cell Signaling Technology (Danvers, MA). Anti-β-actin, and all other reagents were obtained from Sigma-Aldrich (St. Louis, MO).

### Cell Line

Human pancreatic cancer (Panc-1 and BxPC3) cells were purchased from the American Type Culture Collection (ATCC, Manassas, VA), mouse pancreatic cancer Panc02 cells was described previously (Wang B. et al., 2001). All cancer cells were maintained in a humidified atmosphere with 5% carbon dioxide at 37°C. Panc-1, Panc02, and BxPC3 cells were routinely cultured in either Dulbecco modified Eagle medium with high glucose or RPMI 1640 medium, respectively (Invitrogen Corp, Grand Island, NY), containing 10% heat inactivated fetal bovine serum ([FBS], Hyclone Laboratories Inc, Logan, UT) supplemented with 50 IU/ml penicillin, 50 μg/ml streptomycin, and 2 mM L-glutamine from GIBCO (Invitrogen). Human pancreatic ductal epithelial cells (HPDE) were cultured in Gibco™ Keratinocyte SFM (Cat# 17-005-042). All cell lines were verified via microscopic morphology check and DNA characterization.

### Cell Proliferation/Growth *in vitro*

Human or mouse pancreatic cancer cells (1 × 10^4^) were seeded in 96-well plates in triplicates. After incubation for 16–24 h, cells were exposed to various concentrations of Fraction B (in 0.5% serum containing medium). After an additional 72 h, the cell growth was assessed by either counting the viable cells with Vi-CELL XR (Beckman Coulter, Brea, CA) or MTT assay (Yang et al., 2009).

Human pancreatic epithelial cells (1 × 10^4^) were plated in 96-well plates. After incubation for 16 h, cells were treated with Fraction B in the normal cell culture medium as described in the previous section. After an additional 72 h, cell growth was determined by PrestoBlue Assay (Yang et al., 2019).

For analysis of sphere growth of Panc-1 cells, Panc-1 cells were cultured in tumorsphere culture medium of DMEM/Nutrient Mixture F-12 Ham medium containing 20 ng/ml recombinant EGF, 10 ng/ml recombinant basic fibroblast growth factor, 2% N2, and 1% B27 supplements (Life Technologies) for 2–4 weeks. The typical primary spheres with >50 cells isolated from the culture spheres was digested in StemProAccutase (Life Technologies) for a single-cell suspension and plated for secondary spheroid colony formation assay in tumorsphere culture medium. They were then treated with different doses of Fraction B (0.25–1 mg/ml). Seven days after the treatment, each well was examined using a light microscope and total numbers of spheroid colonies were counted.

### Cell Cycle and Apoptosis Analysis

For cell-cycle analysis, cells (2.5 × 10^5^) cultured in 6-well plates were treated with Fraction B (0.5, 1, and 2 mg/ml) for 24 h. Cells were then trypsinizied and centrifuged. The cell pellets were suspended, washed with 1X phosphate-buffered saline solution (PBS), and were fixed in 70% ethanol at 4°C overnight. The fixed cells were further washed with 1X PBS and were suspended in PBTB staining solution containing PBS, propidium iodide (10 μg/ml), 0.5% bovine serum albumin, 0.005% Tween-20, and DNase-free Rnase (1 μg/ml). Cells were incubated in the dark for 30 min at 37°C prior to fluorescence-activated cell-sorting analysis (FACS) using a BD FACS Caliber flow cytometer (BD Biosciences, San Jose, CA). The percentage of cells in each phase of the cell cycle was determined from the DNA histogram content.

The apoptotic cell was further measured by Annexin V surface staining in Panc-1 cells treated with Fraction B (0.25–1 mg/ml) for 24 h. Briefly, cells (2.5 × 10^6^) were double-stained with fluorescein isothiocyanate–conjugated Annexin V and propidium iodide per the manufacturer’s instructions (BD Biosciences). Fluorescence was detected by a BD FACS Caliber flow cytometer and was analyzed using CellQuest^TM^ Pro software (BD Biosciences).

### LC/MS/MS Analysis

Glycolysis and glutamine metabolites were extracted in a manner similar to previously published methods (Cascone et al., 2018)**.** An Agilent 1200 HPLC system coupled with 6,460 Triple Quad MS detector was used to quantify citrate, isocitrate, 2-hydroxyglutarate, α-ketoglutarate, malate, and fumarate analysis. Six analytes were chromatographically separated on a Phenomenex Synergi Hydro RP column (2.5 µm, 2.0 × 50 mm) using 2-hydroxyglutarate-d3 as an internal standard. Mobile phase was 0.1% formic acid in water and a flow rate was 0.2 ml/min. The individual metabolite was detected using electrospray negative ionization and MRM monitoring the transitions at m/z 191→111 for citrate, m/z 191→111 for isocitrate, m/z 147→129 for 2-hydroxyglutarate, m/z 144.9→57.1 for α-ketoglutarate, m/z 133→ 115 for malate, m/z 115→ 71 for fumarate, and m/z 150→132 for 2-hydroxyglutarate-d3. Similarly, Agilent 1200 HPLC system coupled with 6,460 Triple Quad MS detector was used to detect lactate, pyruvate, glutamine and glutamate. Four analytes were separated on an Agilent XDB C18 column (5 μm, 4.6 × 150 mm) using 2-hydroxyglutarate-d3 as internal standard. Mobile phase A was 0.1% formic acid in water and mobile phase B was 0.1% formic acid in acetonitrile. Lactate, pyruvate, glutamine and glutamate were eluted using a gradient, starting at 5% of B at 0 min, held for 1 min, and changed to 10% of B at 6 min, held for 1 min, then changed to 90% of B at 7.1 min and held until 12 min, then changed back to 5% of B at 12.1 min. The flow rate was 0.3 ml/min. These four metabolites were also detected using electrospray negative ionization and MRM monitoring the transitions at m/z 89→43 for lactate, m/z 87→43 for pyruvate, m/z 146→102 for glutamate, and m/z 145→127 for glutamine. The protein concentration was examined using a Bradford protein assay (Bio-Rad). The results were normalized by protein and presented as ng/mg protein per tumor tissue.

### Reverse Phase Protein Array

Panc-1 tumor tissues harvested from mice after receiving Fraction B treatment at a dose of 0 and 6 mg/kg were divided into small sections and immediately snap frozen in liquid nitrogen, and tumor lysates were subjected to reverse phase protein array (RPPA) analysis by the Functional Proteomics Core Facility at The University of Texas MD Anderson Cancer Center as described previously (Tibes et al., 2006; Hennessy et al., 2010).

### Histopathology and Immunohistochemistry

Formalin fixed tumor tissues were paraffin embedded and processed for the identification of cell proliferation, apoptosis and CD44 expression by immunohistochemistry (IHC) staining. For IHC staining, slides were baked at 60°C for over 2 h and then deparaffinized and rehydrated. Antigens were unmasked by heat induced antigen retrieval. Slides were then immersed in 3% H_2_O_2_-Methonal solution followed by blocking with 5% goat serum in 0.3% Triton X-100 PBS. Slides were stained with Ki-67, Cleaved Caspase 3, or CD44 antibody in a humidified chamber overnight at 4°C. Slides were washed with PBS for three times followed by incubation with secondary antibody for 45 min at room temperature. Slides were incubated with ABC (Vector Laboratories, Burlingame, CA) followed by DAB substrate for antibody visualization and counterstained with Mayer’s hematoxylin, dehydrated, and mounted with ClearMount Mounting Medium (American MasterTech, Lodi, CA). The stained slides were scanned with Aperio AT2 bright-filed slide scanner and tissue sections were quantified for IHC staining with Aperio image analysis algorithms. The staining result was determined by the percentage of positive cells.

### Immunoblotting

Panc-1 cells were treated with Fraction B for 24 h or 48 h and then were harvested in sodium dodecyl sulfate buffer. Total protein (50 μg per well) was subjected to separation on a 10–15% sodium dodecyl sulfate gel and then was transferred onto polyvinylidene fluoride membranes. The membranes were blocked in Tris-buffered saline solution with 5% nonfat milk and then were probed with primary antibodies for CD44 (No. 357259, Cell Signaling, Danvers, MA), CD133 (No. 5860, Cell Signaling), Met (No. 8198, Cell Signaling) and LDHA (No. 3582, Cell signaling) at 4°C overnight. Membrane was incubated in IRDye Secondary antibody (1:20,000, No. 925–32211, Li-COR) for 1 h at room temperature followed by three washes with TBS-T. Membrane was imaged with LiCor Odyssey scanner (LI-COR Biosciences, Lincoln NE) and bands were quantified with Image Studio Lite software. Β-actin was used for normalization of results.

### Animal Models of Tumorigenesis

Our animal studies were conducted according to The University of Texas MD Anderson Cancer Center Animal Care and Use Committee rules and regulations (IACUC protocol number: 00000669-RN02). The animals were housed in facilities approved by the Association for Assessment and Accreditation of Laboratory Animal Care International in accordance with the current regulations and standards of the United States Department of Agriculture and Department of Health and Human Services. For tumorigenesis of human Panc-1 cells, pathogen-free female athymic BALB/c nude mice (6–8 weeks old) with body weight 25 ± 5 g, purchased from the National Cancer Institute, were injected with intrapancreatic injection of Panc-1 cells at 1 × 10^6^ cells/0.1 ml in Hank’s balanced salt solution per mouse. Ten days after injection, all animals that received orthotopic tumor cells inoculation were imaged using an MR Scanner in the Small Animal Imaging Facility (SAIF, M. D. Anderson Cancer Center, TX) applying a standard protocol for pancreatic cancer imaging in mice. Tumor bearing mice were randomly assigned to vehicle control (physiological saline) or Fraction B treatment groups, when tumor volume reached 72.3 ± 0.7 (mm^3^) by MRI examination. Fraction B was administered via IP injection daily for 2 weeks with a dose of 3 or 6 mg/kg animal body weight. For Panc02 cell tumorigenesis, C57BL/6 mice were injected with Panc02 cells (5 × 10^5^ cells/mouse) subcutaneously on the right flank and then randomly assigned to vehicle control and Fraction B (6 mg/kg animal body weight) treatment when tumor volume reached 50 mm^3^. Tumor volume (mm^3^ = ½ × long diameter × short diameter^2^) was measured and calculated every other day. At the end of the treatment, mice were euthanized, and the tumors were collected and either fixed in a 10% formalin-PBS solution or flash frozen in liquid nitrogen and stored at −80°C for further analysis.

### Blood CBC and Chemistry Analysis

At the end of treatment, blood was collected by cardiac puncture from mice bearing Panc-1 tumor. They were then subjected to CBC differential and blood chemistry analysis by the histopathological core of the Department of Veterinary Medicine and Surgery at the University of Texas MD Anderson Cancer Center using their standard operation procedure.

### Statistical Analysis

GraphPad Prism was used for the statistical analysis (*t*-test or ANOVA). *p*-values less than 0.05 were considered statistically significant. Data are presented as the mean ± SD.

## Results

### Fraction B Inhibits Proliferation of Human and Mouse Pancreatic Cancer Cells *in vitro*

**FIGURE 1:**
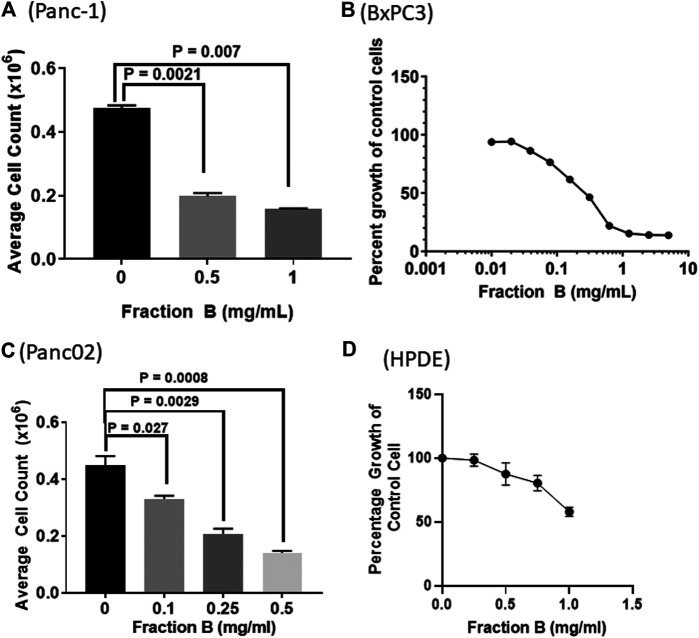
The dose dependent effects of Fraction B on pancreatic cancer and normal cell proliferation. The cells were treated with Fraction B for 72 h. Human pancreatic cancer **(A)** Panc-1 **(B)** BXPC3, and **(C)** mouse pancreatic cancer Panc02 cells as well as human pancreatic ductal epithelial cells **(D)**. The cell growth was measured by cell counting and cell viability was assessed by MTT or PrestoBlue assay. All data are expressed as mean ± SD.

To understand the role of Fraction B on the proliferative activity of the pancreatic cancer cells, we determined the action of Fraction B on the proliferation of human pancreatic cancer Panc-1 and BxPC3 cells, and mouse Panc02 cells by counting viable cell number using hemocytometer and Vi-CELL XR cell counter (Beckman Coulter, Brea, CA) or MTT assay. We found that Fraction B treatment dose-dependently inhibited cell proliferation with very similar potency in all three cell lines tested with IC_50_ at 200—400 μg/ml, suggesting that the anti-proliferative effect of Fraction B in pancreatic cancer is general and not cell-line specific (Figures 1A–C). In contrast, when the immortalized human pancreatic epithelial cells were treated with similar concentration of Fraction B, Fraction B even at 1 mg/ml concentration did not cause 50% of cells death (Figure 1D), suggesting Fraction B selectively inhibited the proliferation of pancreatic cancer cells.

### Fraction B Treatment Promotes Cell Cycle Arrest and Causes Apoptosis in Panc-1 and Panc02 Cells

**FIGURE 2:**
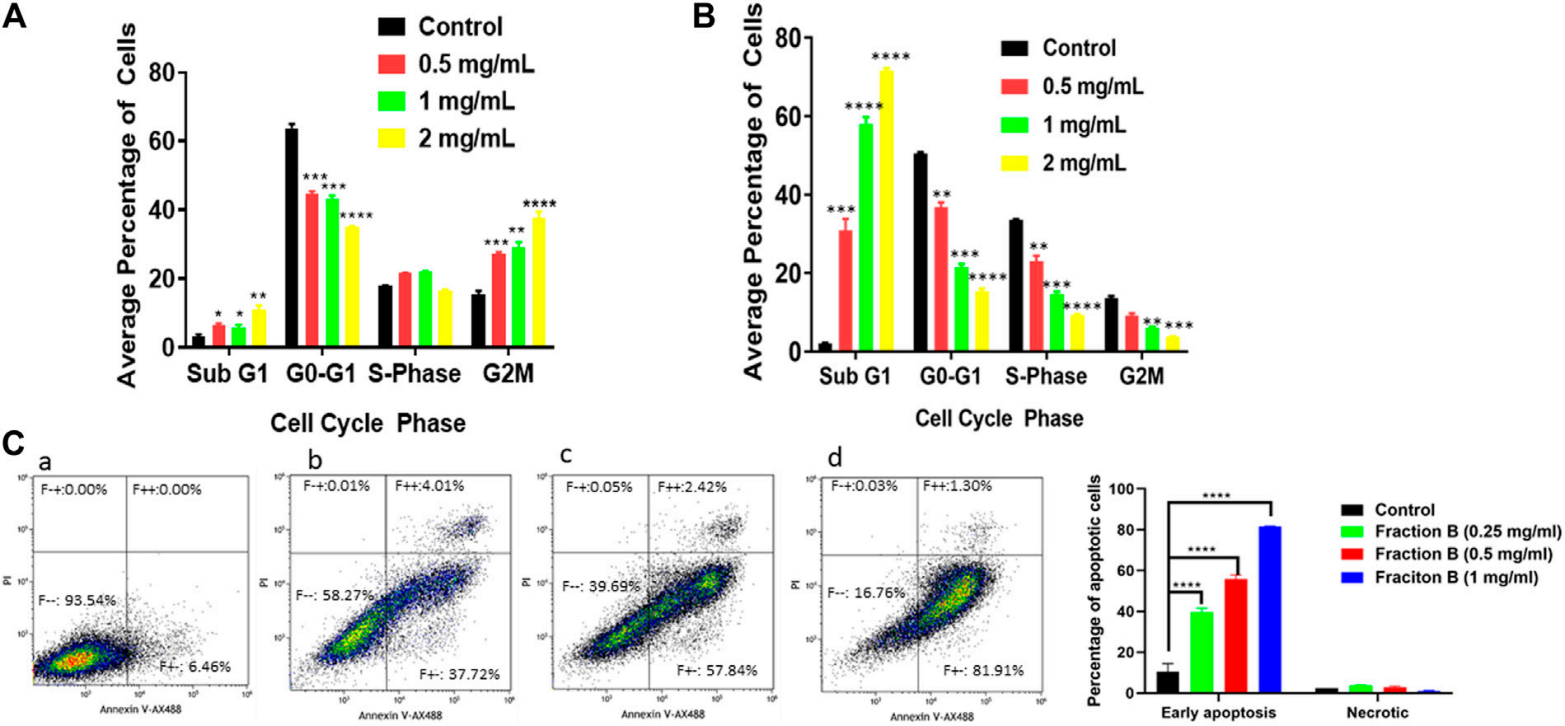
Impact of Fraction B treatment on cell cycle profile and apoptosis in pancreatic cancer cell lines. Pancreatic cancer cells were treated with different concentrations of Fraction B for 24 h. Cell cycle **(A, B)** and apoptotic **(C)** analysis show a dose dependent effect **(A, C)** in Panc-1 cells **(B)** Panc02 cells treated with Fraction B. (a). Vehicle control; (b). Fraction B (0.25 mg/ml); (c). Fraction B (0.5 mg/ml); (d). Fraction B (1 mg/ml). All data are expressed as mean ± SD. **p < 0.01, ***p < 0.001, ****p < 0.0001 treated versus vehicle control group.

Next, we addressed the effect of Fraction B on cell cycle progression. We applied the Fraction B to Panc-1 and Panc02 cells for 24 h, then they were subjected to flow cytometry cell cycle analysis using PI staining. We found that Fraction B (500 μg/ml) significantly increased subG1/G0 phase cells by 2-fold and 25 fold in Panc-1 cells (Figure 2A) and Panc02 cells (Figure 2B) respectively, compared to that of vehicle treatment (*p* < 0.01 and <0.001, respectively). Fraction B-treated Panc-1 cells had more apoptotic cells than the control samples, as quantitated by Annexin V staining (*p* < 0.0001) (Figure 2C). We also found that Fraction B treatment led to significant G2/M cell cycle phase arrest in Panc-1 cells. The induction of apoptosis and alteration of cell cycle by Fraction B in these cells were in a dose-dependent manner, suggesting that cells treated with Fraction B were undergoing apoptosis and/or cell cycle arrest.

### Fraction B Inhibits Tumor Growth in Mouse Models *in vivo*

**FIGURE 3:**
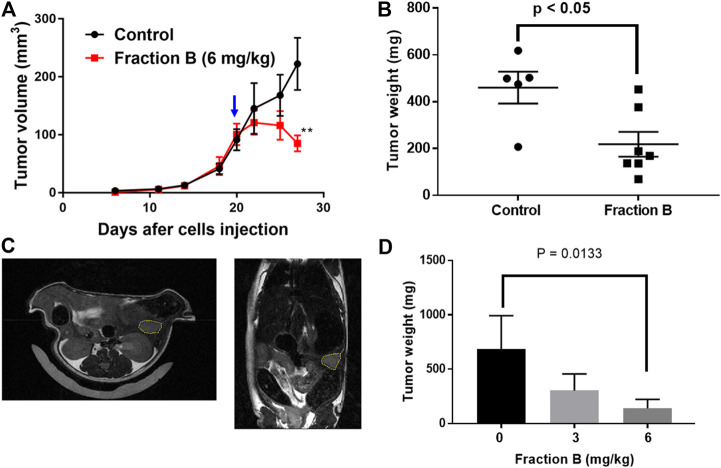
Growth inhibitory effect of Fraction B on mouse and human pancreatic tumor models. The syngeneic mouse tumor model was developed by injecting mouse Panc02 cells into C57BL/6 mice. The growth curve of Panc02 derived tumor development shows **(A)** tumor volumes **(B)** terminal tumor weight of Panc02 after being treated with Fraction B. Human pancreatic cancer cell Panc-1 mouse orthotopic tumor model was developed by injecting Panc-1 cells into the head of mouse pancreas. The tumor size was determined by MRI imaging 10 days after Panc-1 cells were injected **(C)**. **(D)** terminal weight of Panc-1 derived tumor shows the inhibitory effect of Fraction B (6 mg/kg). Data are presented as mean ± SE (*n* = seven to eight per group). **p < 0.01 treated versus vehicle control.

We further evaluated the efficacy of Fraction B on pancreatic cancer, by conducting an antitumor efficacy study in mouse pancreatic cancer Panc02 syngeneic model and human Panc-1 orthotopic mouse model. Each group of mice were subcutaneously injected with Panc02 cells (5 × 10^5^) to the right flank of the mice. Fraction B was administered via IP injection daily for 2 weeks in the Panc02 syngeneic model. As shown in Figure 3, Fraction B significantly suppressed the growth of Panc02 tumor (*p* < 0.01). In fact, Fraction B led to tumor regression after two weeks of treatment (Figure 3A). The average terminal tumor weight of mice treated with Fraction B (6 mg/kg) was 218.1 ± 53.1 mg which was 53% smaller than that of control group (460.2 ± 68.0 mg) (*p* < 0.05) (Figure 3B). Next, we further evaluated the activity of Fraction B on tumor development under physiologically relevant environment. To address that, we injected human pancreatic cancer Panc-1 cells (1 × 10^6^) directly in the head of the pancreas and monitored the effect of treatment of Fraction B on tumor growth. The tumor size was determined by MRI imaging (Figure 3C) 10 days after tumor cells were injected to the head of pancreas and mice were then randomized to either control or treatment group based on calculated tumor volume via MRI imaging. As shown in Figure 3D, the average tumor weight of mice treated with Fraction B (6 mg/kg) was 139.5 ± 68.0 mg, which was 80% significantly smaller than that of control group (686.1 ± 306.7 mg) (*p* < 0.05). Together, this data strongly suggests that Fraction B is highly efficacious against the development of pancreatic cancer.

### Reverse Phase Proteomic Array Analysis of Fraction B Treated Tumor Tissue in Orthotopic Pancreatic Tumor Model

**FIGURE 4:**
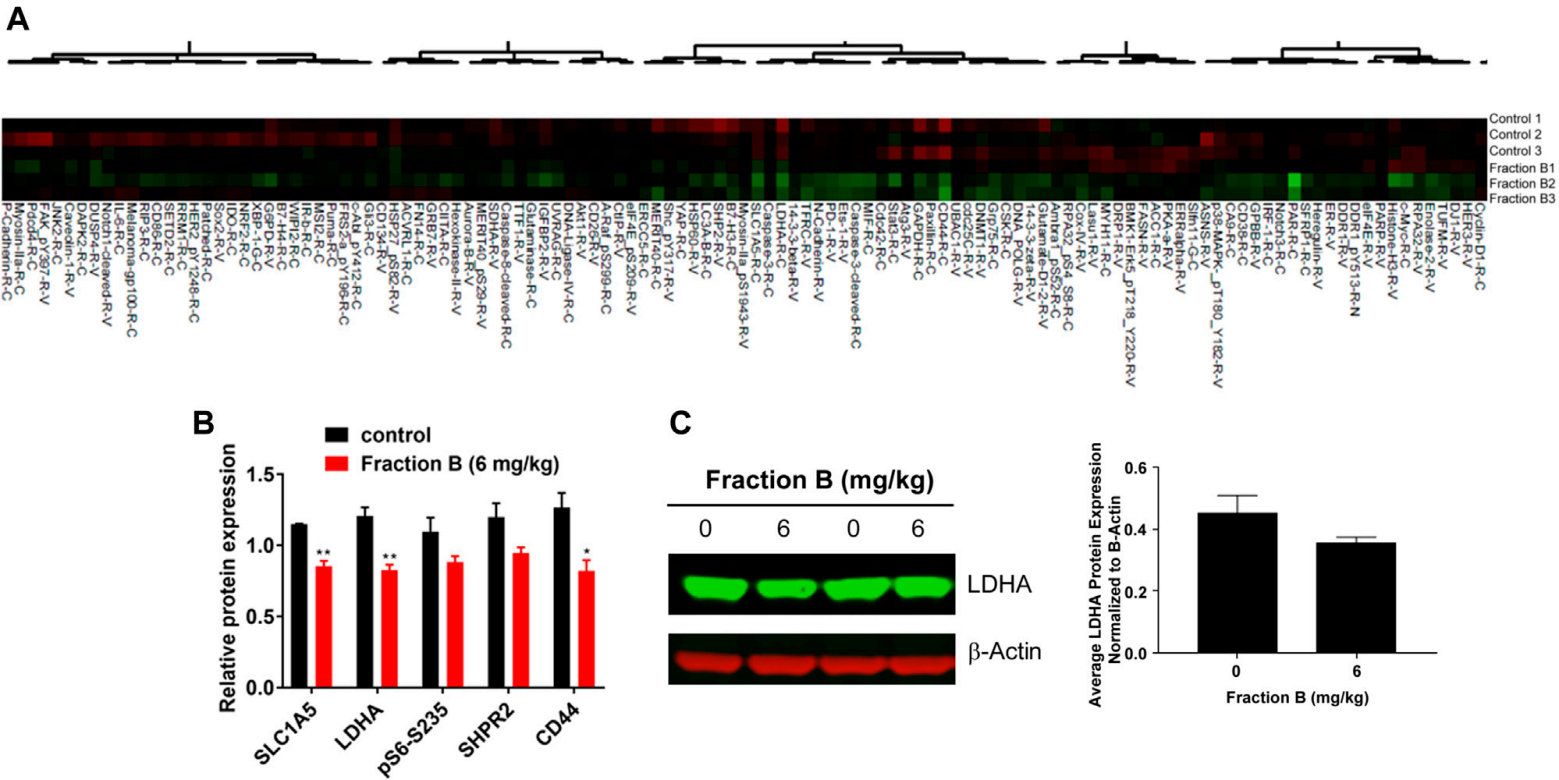
Identification of Fraction B target proteins by Reverse Phase Proteomic Array (RPPA) analysis: RPPA analysis was done by comparing Panc-1 tumor derived tissue either treated with Fraction B (6 mg/kg) or Vehicle. **(A)** Heatmap of the proteins involved in cell growth, cell signaling and cell metabolism. **(B)** Fraction B significantly suppressed the expression of CD44, LDHA and SLC1A5 in Panc-1 tumor tissues compared to that of vehicle control. **(C)** Western blot of LDHA protein in Panc-1 tumor tissues. Data are presented as mean ± SD. *p < 0.05, **p < 0.01 versus vehicle control.

To understand the potential mechanism involved in Fraction B-elicited tumor volume reduction, we performed Reverse Phase Proteomic Array (RPPA) analysis using Panc-1 tumor tissue treated with Fraction B compared to that of controls. Among numerous proteins affected by Fraction B, treatment (6 mg/kg) significantly down-regulated CD44 by 54%, a known stem cells marker, LDHA (32%), a key glycolytic enzymes, SLC1A5 (26%), a glutamine transporter (Figures 4A,B). The down regulation of LDHA protein by Fraction B in Panc-1 tumor tissues was further confirmed by Western blot analysis (Figure 4C). These findings suggest that antitumor activity elicited by Fraction B may be due to suppression of cancer stemness (reduction in CD44 expression) and reducing energy related metabolic pathways (LDHA, SLC1A5).

### Evaluation of the Inhibitory Effect of Fraction B on Pancreatic Cancer Stem Cells

**FIGURE 5:**
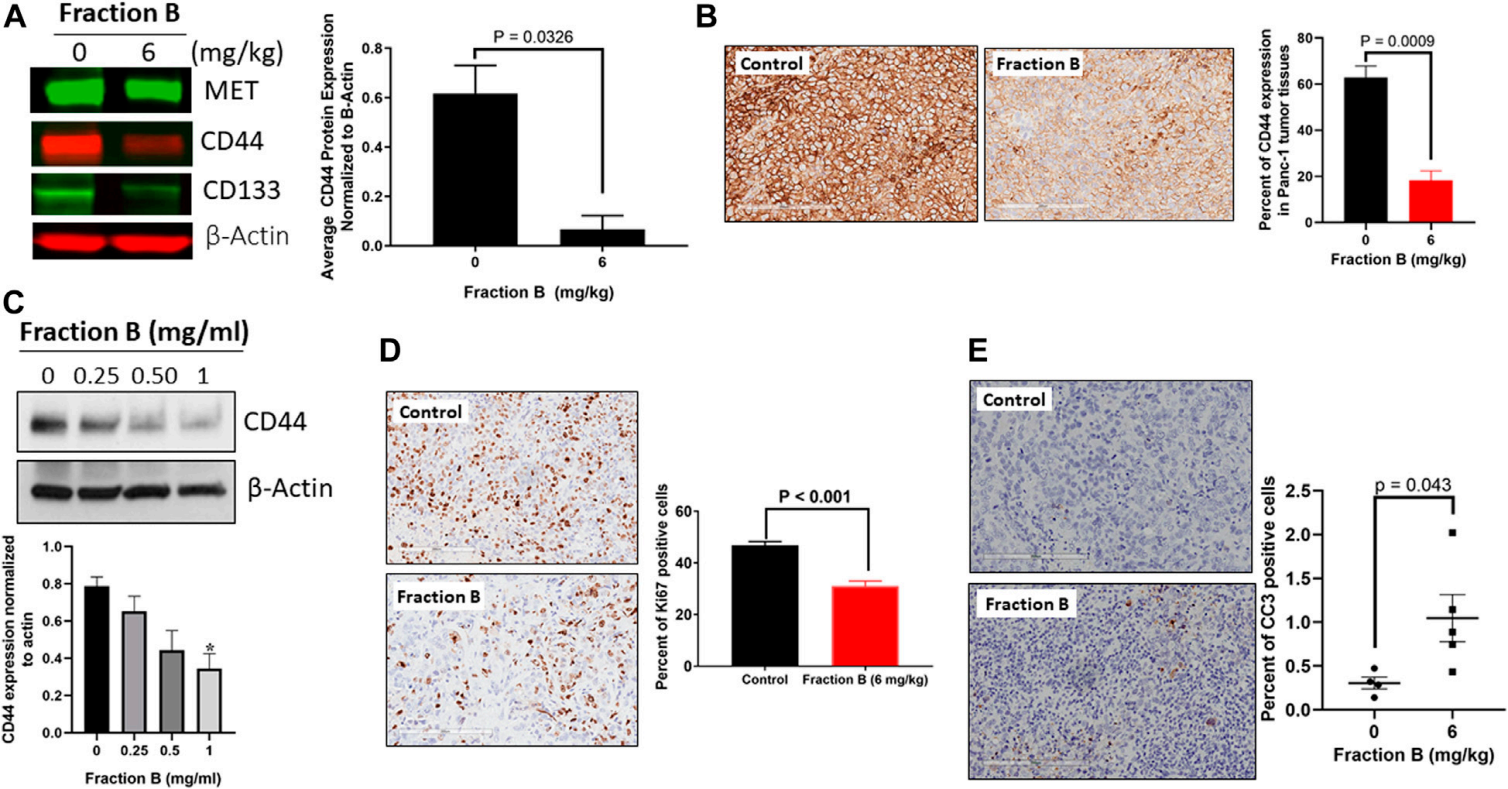
Inhibitory effect of Fraction B on cancer stem cell markers expression and cell proliferation and induction of apoptosis in Panc-1 derived tumor. **(A)** Western blot analysis of key stem cell marker CD44 in Fraction B (6 mg/kg) treated Panc-1 tumor tissues. **(B)** IHC staining of CD44 protein in Panc-1 tumor tissues. **(C)** Western blot analysis of CD44 protein expression in Panc-1 cells treated with Fraction B for 24 h. **(D)** Ki67 staining of Panc-1 tumor tissues. **(E)** Cleaved caspase 3 (CC3) staining of Panc-1 tumor tissues. Data are presented as mean ± SE. *p < 0.05 versus vehicle control.

**FIGURE 6:**
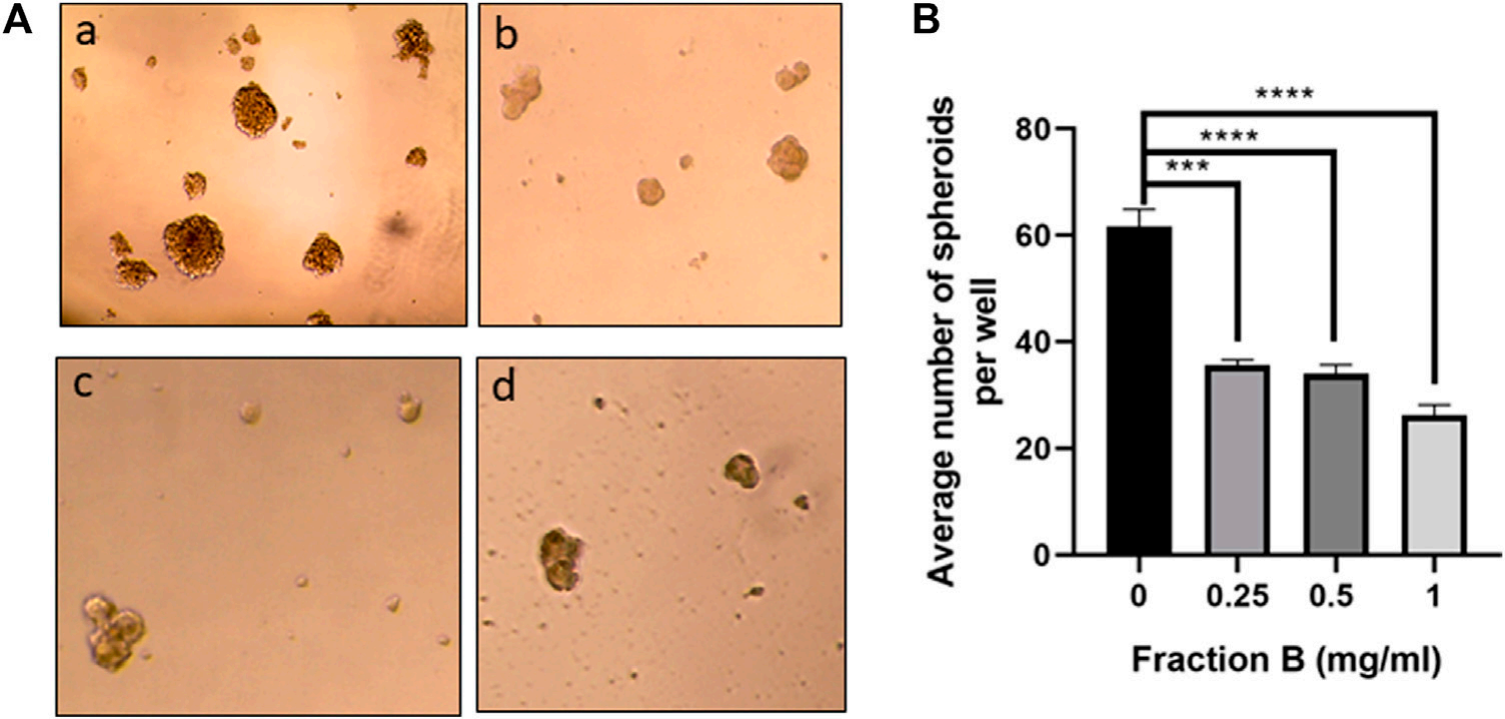
Fraction B inhibited Panc-1 self-renewal capacity. **(A)** Panc-1 spheroid forming cells were treated with Fraction B at 0.25 (b), 0.5 (c), and 1 mg/ml (d) for 7 days and spheroid formation in the control (a) or Fraction B (b–d) treated wells was determined. Pictures were taken at 10x. **(B)** Quantitative assessment showed that Fraction B treatment significantly reduced spheroid formation compared to the control groups. Data are presented as mean ± SD.***p < 0.001, ****p < 0.0001 versus vehicle control.

To further validate the action of Fraction B on pancreatic cancer stemness, we first examined the expression of pancreatic cancer stem cell markers, including CD44, CD133 and Met in Panc-1 tumor tissues treated with Fraction B. As shown in Figure 5A, Fraction B (6 mg/kg) treatment indeed significantly inhibited CD44 expression in Panc-1 tumor tissues (*p* < 0.05). Interestingly, we found that Fraction B treatment also suppressed the expression of CD133 and Met by 57 and 24%, respectively compared to that of vehicle control, but the difference was not statistically significant (Supplementary Figure S1). IHC staining CD44 protein in Panc-1 tumor tissues treated with Fraction B showed that the percentage of CD44 expression in Panc-1 tumor tissues was notably reduced by 71.4% compared to that of control tissues (*p* = 0.0009, Figure 5B). We then performed a dose-dependent treatment of Fraction B and observed CD44 expression in Panc-1 cells. The abundance of CD44 protein in Panc-1 cells was dose-dependently reduced after being treated with Fraction B for 24 h with statistically significant inhibition of CD44 in Fraction B (1 mg/ml) treated cells as opposed to that of vehicle control (*p* < 0.05) (Figure 5C *).* Western blotting showed that Fraction B treatment inhibited CD44 expression and correlated with reduced Ki67 expression (*the percentage of Ki67 positive cells in Fraction B treated vs control tumor = 31.0* ± *2.1% vs. 46.8* ± *1.4%*), the nuclear marker for cell proliferation in Panc-1 tumors formed in mice (Figure 5D) and significant 2.5 fold increases in cleaved caspases 3, an apoptotic cell markers, in fraction B treated Panc-1 tumor compared to that of control group (Figure 5E). To further determine whether Fraction B could affect the function of cancer stem cells, we examined the self-renewal capacity of Panc-1 stem cells after being treated with Fraction B. As shown in Figure 6, the number of Panc-1 spheroids formed in Fraction B treated cells was significantly less compared to that of vehicle controls (*p* < 0.0001). These data suggest that Fraction B inhibited the tumor cell proliferation and caused induction of apoptosis potentially through suppressing the stemness of pancreatic cancer cells.

### Fraction B Treatment Markedly Alters Glycolysis and Glutamine Metabolism in Panc-1 Tumor

**FIGURE 7:**
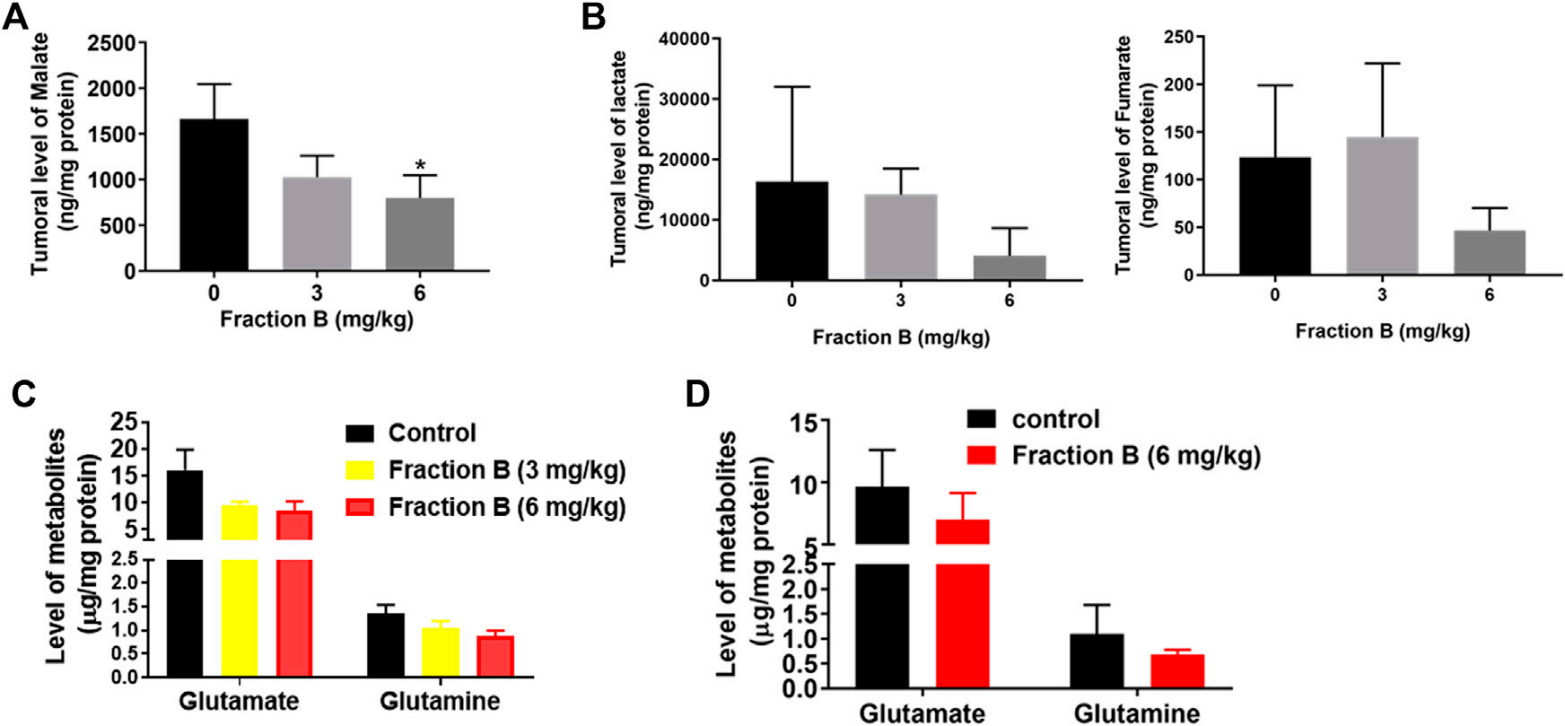
Alterations of critical CSC related metabolic pathways in tumors by Fraction B treatment. Changes of glucose metabolites **(A)** malate, **(B)** lactate and fumarate, and glutamine metabolites **(C)** in Panc-1 tumors. **(D)** Glutamine metabolites in Panc02 tumors. Data are presented as mean ± SE. *p < 0.05 treated vs control.

CSCs have distinct metabolic traits in comparison to non-CSCs as indicated by elevated glucose consumption and lactate synthesis in CSCs (De Francesco et al., 2018; Peixoto and Lima, 2018) and noncanonical glutamine pathway is crucial for tumor growth and balance of oxidative stress in pancreatic CSCs (Son et al., 2013; Li et al., 2015; Snyder et al., 2018). Since Fraction B down-regulated protein abundance of LDHA, the enzyme responsible for conversion of pyruvate to lactate and SLC1A5, a glutamine transporter protein, we then examined the level of glycolysis metabolites and glutamine metabolites using the sensitive and specific analytical method developed in our lab using LC/MS/MS instrument. LC/MS/MS analysis confirmed that Fraction B significantly reduced glucose metabolites involved in glycolysis and TCA cycle, including lactate and malate in Panc-1 tumor tissues compared to that of control tumor tissues (Figures 7A,B). The level of fumarate was also lower in the Fraction B treated Panc-1 tumor tissues than that of control group, but the difference was not statistically significant (Figure 7B *, right*). Tumoral levels of glutamate and glutamine in Fraction B (6 mg/kg) treated mice were 47 and 36%, respectively lower than those from control groups (Figure 7C), suggesting Fraction B dose-dependently reduced both glycolytic and glutamine metabolism in human Panc-1 tumor. In contrast, only the levels of glutamate and glutamine, but not glycolytic metabolites, were reduced by 28 and 32%, respectively, in Fraction B treated Panc02 tumor (Figure 7D), suggesting the impact of metabolic changes elicited by Fraction B in Panc-1 and Panc02 tumor is different. Whether it is caused by the differences of genetic alterations between the two cell lines deserves further investigation.

### Fraction B Treatment Posed No Side Effects in Mouse Bearing Panc-1 Tumor

While Fraction B showed promising antitumor efficacy in both mouse Panc02 and human Panc-1 tumor model, it would be logical to question how safe this particular product is for mice despite not seeing any body weight changes in the mice treated with Fraction B compared to that of control mice (Supplementary Figure S2). We therefore collected blood from the Panc-1 tumor bearing mice and subjected it to CBC and blood chemistry analysis. The results of CBC and blood chemistry analysis by the pathological core facility at the Department of Veterinary Medicine and Surgery at MDACC showed that Fraction B did not cause any substantial changes on blood CBC account (Table 1), and liver and kidney functions (Table 2), suggesting Fraction B is relatively safe to be administered. In light of such pronounced inhibitory effect of Fraction B in the treatment of Panc-1 tumor with very limited toxicity and lack of effective treatment for pancreatic cancer, Fraction B may have translational value for PDAC treatment.

**TABLE 1:** CBC count of Panc-1 bearing mice treated with Fraction B.

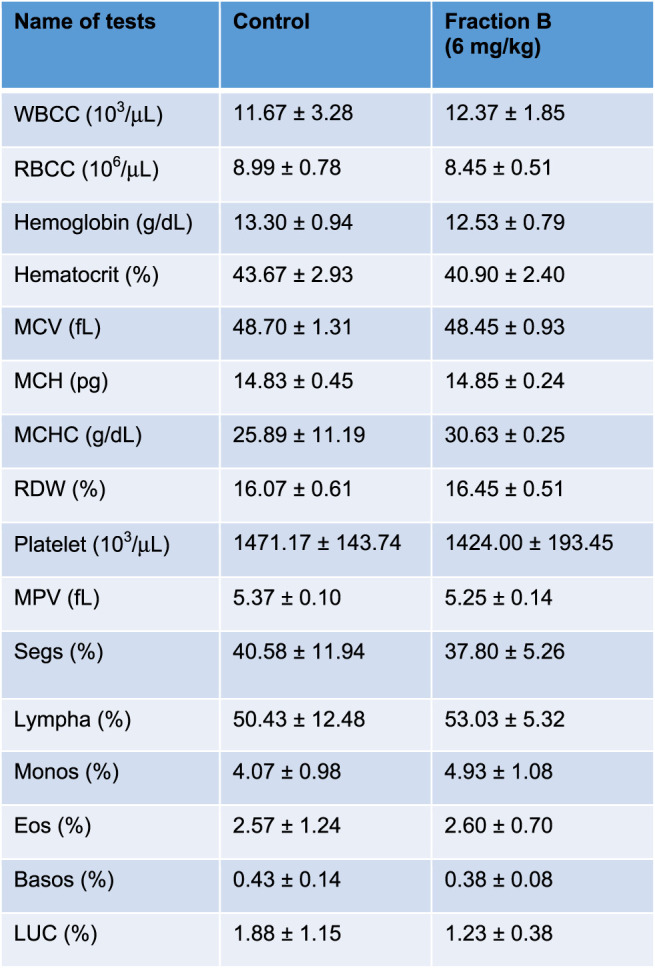

**TABLE 2:** Blood chemistry of Panc-1 bearing mice treated with Fraction B.

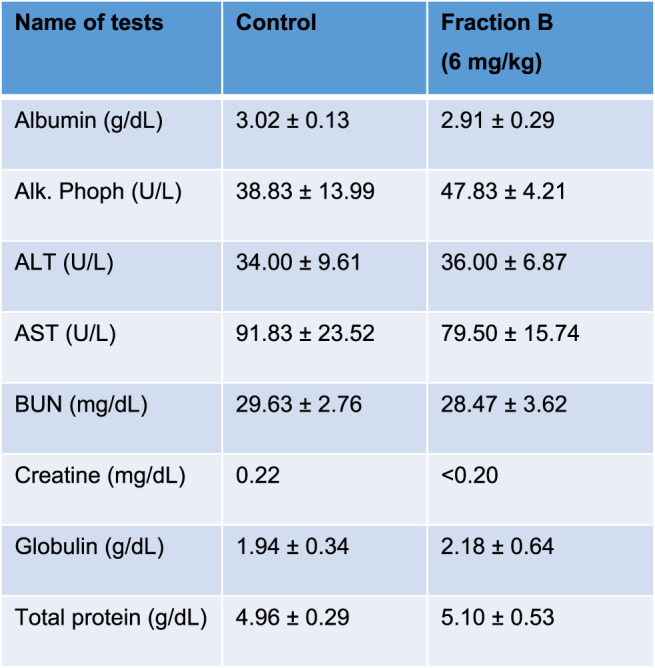

## Discussion

PDAC is the most aggressive disease; the mortality rate has been almost the same over the last three decades. Novel and effective therapeutic strategies are desperately needed to improve the outcomes of patients with PDAC. Though a large number of phase II/III studies have been conducted in the past decades, successful treatment options remain limited (Ryan et al., 2014). In this study, we provided several lines of evidence to demonstrate the potent anti-tumor effects of Fraction B in PDAC. These are: 1) Fraction B treatment *in vitro* significantly inhibits PDAC cell growth; 2) Fraction B treatment *in vitro* dose-dependently induces PDAC cell apoptosis; 3) Administration of Fraction B significantly suppresses established PDAC tumor growth in mouse models of both mouse and human PDAC; 4) Mechanistically, Fraction B treatment significantly inhibit PDAC stemness features including reducing the stem cell self-renewal capacity, suppressing CSC related marker CD44, CD133, and Met expression and interfering in CSC related metabolic profiles; 5) Administration of Fraction B in human PDAC tumor bearing mice shows no or very limited systemic toxicity. To our knowledge, this is the first study to show potent anti-PDAC effect of the Fraction B derived from catfish skin preparation with remarkable multiple scientific and clinical implication.

The devastating features of pancreatic cancer are its early metastasis and resistance to currently available chemo-radio/therapies, largely attributed to CSCs (Li et al., 2007). Targeting CSCs has been the hotspot in pancreatic cancer research and holds a great promise to improve the outcomes of patients with PDAC. One of the exciting findings in this study is that Fraction B treatment can significantly inhibit PDAC stemness, which was substantiated by Fraction B dose-dependently inhibiting CD44 expression. Since CD44 protein is not only solely functions as a CSC cell surface marker, but also profoundly regulates cancer stemness through its cleaved intracellular domain- CD44-ICD DNA binding to directly regulate CSC related gene expression or interaction with other critical stemness factors supporting their functional activation (Cho et al., 2015). It is therefore not surprising to find the suppressive action of Fraction B reflected on the expression of CD133 and Met, which were previously reported as PDAC CSC markers (Li et al., 2011; Herreros-Villanueva et al., 2012). It should be noted that the inhibitory effect of Fraction B treatment on CD44 expression was initially identified through the unbiased RPPA analysis using human PDAC xenograft tumor tissue samples and was then further validated *in vitro* using PDAC cell line, suggesting that CD44 might be a true target of Fraction B; however, the detailed molecular mechanisms by which Fraction B inhibits CD44 await for further study.

Another very interesting finding of this study is the significant effect of Fraction B on glucose and amino acid metabolisms in the tumor tissues, with drastic suppression of glycolysis and glutamine consumption. The glycolytic energetics under mitochondrial respiratory suppression together with activation of the pentose phosphate pathway (PPP) in cancer cells reduce cellular production of reactive oxygen species (ROS), promote tumor growth and support the maintenance of the stem cell phenotype conferring therapeutic resistance (Peixoto and Lima, 2018). CD44 interacts with pyruvate kinase M2 (PKM2) and hence enhances the glycolytic phenotype of cancer cells that are either deficient in p53 or exposed to hypoxia (Tamada et al., 2012). PDAC cells are addict to glutamine metabolism pathway for redox homeostasis, in which oncogenic KRAS mediated upregulation of aspartate transaminase and suppression of glutamate dehydrogenase play a critical role (Son et al., 2013). A CD44 variant (CD44v), highly expressed in PDAC CSC-like cells (Miyatake et al., 2018), interacts with xCT, a glutamate-cystine transporter, enhances capacity for glutathione (GSH) synthesis to defend against reactive oxygen species (ROS), and thereby maintains CSC and stimulates tumor growth (Ishimoto et al., 2011). In this study, we observed that Fraction B not only significantly suppressed the expression of key enzymes responsible for glycolysis, LDHA, and glutamine transporter, SLC1A5, in the tumor tissues, but also reduced the metabolites of glycolysis and glutamine levels. However, whether the significant impact of Fraction B on glycolytic and glutamine metabolic changes in PDAC tumor is mediated through inhibition of CD44 or through other mechanisms still remains unknown. The significant reduction of LDHA and SLC1A5 protein expression in Fraction B treated tumor may represent additional layer of action or regulation and deserves further investigation.

Additionally, we observed that the administration of Fraction B via IP injection induced tumor growth regression in syngeneic mice bearing Panc02 tumors, suggesting that the anti-tumor effect of Fraction B may be involved in the activation of anti-tumor immunity. Fraction B treatment in a diabetic rat model resulted in significant decrease in serum levels of TNF-α, IL-1β, IL-4, and CCL5 and significant increase in IL-2 and IFN-ɣ levels (unpublished data), suggesting that Fraction B may have an immune regulatory function which may favor its antitumor therapy and deserves further investigation. Exploring the underlying mechanisms involved in the regression of Panc02 tumors will be our future research direction. We also realize the limitation of this study, especially the complexity of Fraction B composition, which presents a significant barrier for detailed mechanism study. Further characterization of Fraction B to identify the effective compounds will greatly facilitate the development of novel, effective, and mechanism-based therapeutic strategy that benefits more patients, or at least lead to insight into the role of these components into the different stages of the anti-tumor activities of Fraction B for other anti-cancer research and application. It is worth mentioning that neither the lipid fraction nor the protein fraction of Fraction B each on its own yields the observed biological activities on PDAC (Data not shown). It may be that synergism is involved which requires the involvement of different proteins and lipids at different stages to yield the observed results. Until the stage of enriching the present different cancer treatments with some components of Fraction B is reached, it might be wise to utilize the remarkable curing properties of Fraction B, the natural product of the multicomponent composition.

In brief, we showed for the first time that treatment of PDAC cells with Fraction B preparation from the skin secretions of the catfish could potently suppress the growth of both human and mouse PDAC cells by inhibition of CD44 expression, cancer stemness, and regulation of glucose and glutamine metabolic changes in tumor tissues. Considering the significant anti-tumor effect, while with no or limited systemic toxicity observed in this study, we anticipate that additive or synergistic effect for Fraction B combined with other therapies, particularly immunotherapy and/or radiotherapy for the treatment of PDAC will be worth pursuing. This with better understanding of the mechanistic action of Fraction B promise to revolutionize PDAC treatment and therefore deserve further investigation.

## Data Availability

All original data are presented in the article/Supplementary Material. Further information can be obtained from the corresponding author.
