# Supplementary material for: Fraction B From Catfish Epidermal Secretions Kills Pancreatic Cancer Cells, Inhibits CD44 Expression and Stemness, and Alters Cancer Cell Metabolism
Source: Front Pharmacol. 2021 Jul 19;12:659590. doi: 10.3389/fphar.2021.659590 (PMC8326461; doi:10.3389/fphar.2021.659590)
Supplement: Supplementary file 1 [file Presentation1.PPTX]

## Slide 1
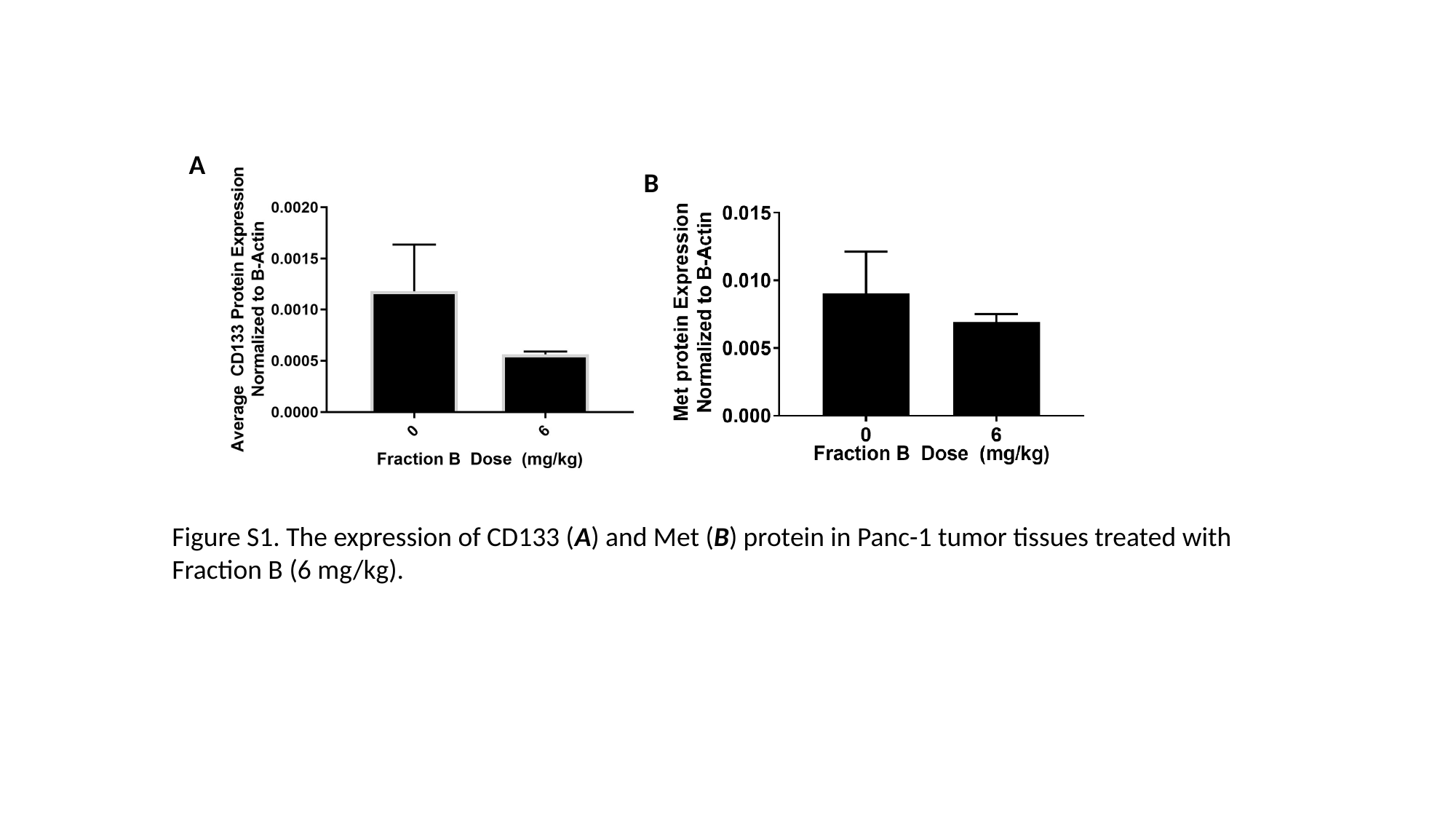

A
B
Figure S1. The expression of CD133 (A) and Met (B) protein in Panc-1 tumor tissues treated with Fraction B (6 mg/kg).

## Slide 2
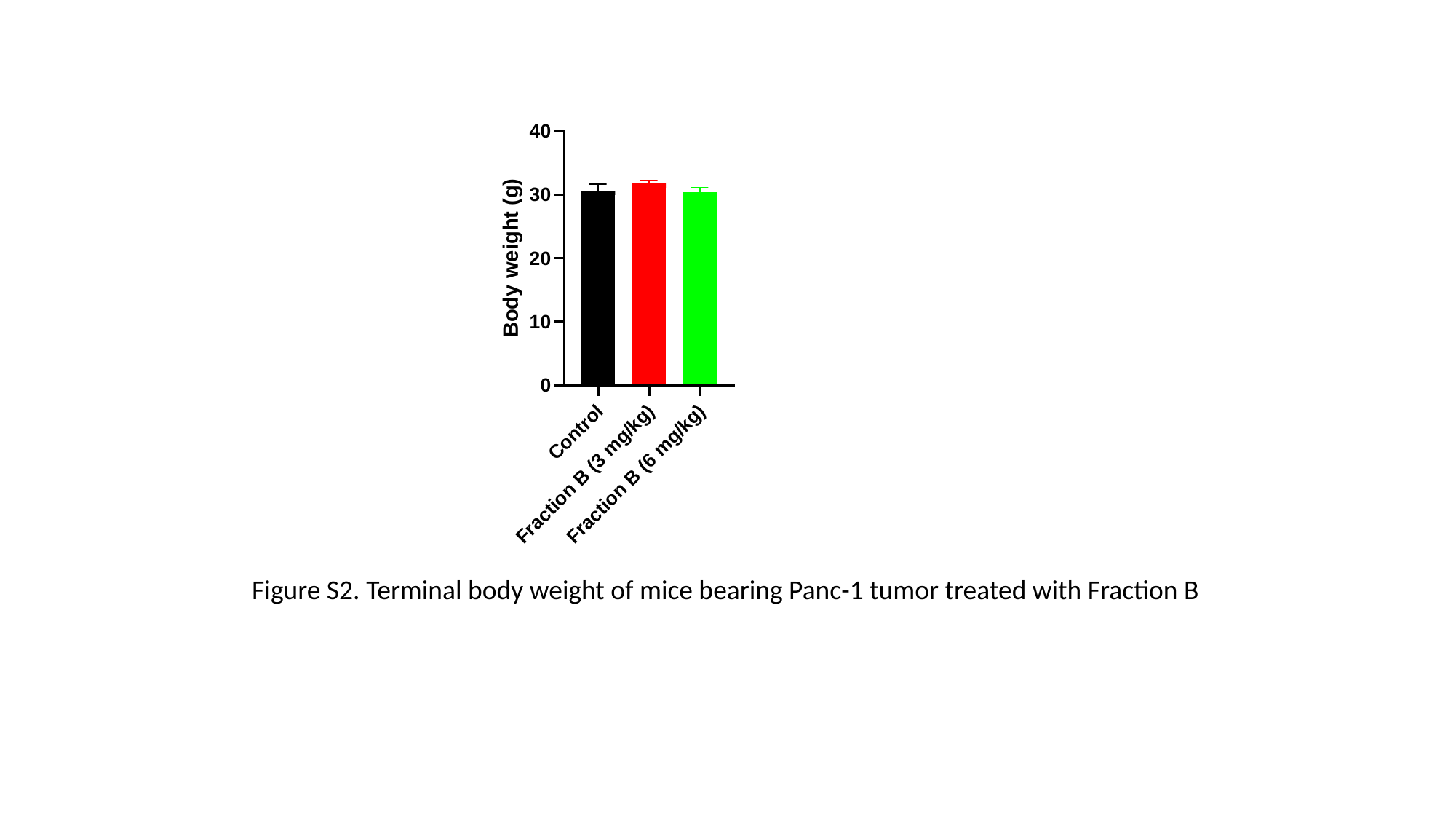

Figure S2. Terminal body weight of mice bearing Panc-1 tumor treated with Fraction B
